# Molecular docking analysis of DprE1 from *M. tuberculosis* with phytochemicals

**DOI:** 10.6026/973206300221754

**Published:** 2026-03-31

**Authors:** Jaynika Raja, J. Jino Blessy

**Affiliations:** 1 Department of Bioinformatics, Sri Ramachandra Faculty of Engineering and Technology, Sri Ramachandra Institute of Higher Education and Research, Chennai, India

**Keywords:** Tuberculosis, decaprenylphosphoryl-β-D-ribose 2'-epimerase (DprE1), Indian phytochemicals, molecular docking, molecular dynamics simulation, nimbolide

## Abstract

Tuberculosis remains a major global health problem due to the emergence of drug-resistant strains of *Mycobacterium tuberculosis*. *Mycobacterium tuberculosis* DprE1, a key enzyme involved in cell wall biosynthesis, was targeted to identify potential anti-tuberculosis agents. Hence, molecular docking and molecular dynamics simulations were performed to screen Indian phytochemicals against DprE1. Among the tested compounds, Nimbolide exhibited one of the strongest binding affinities (-10.3 kcal/mol), outperforming standard antibiotics. Simulation results further confirmed the stability of the DprE1-Nimbolide complex, showing minimal RMSD and RMSF fluctuations over 100 ns. Thus, Nimbolide is as a promising and safe phytochemical candidate for the development of new anti-tuberculosis therapeutics.

## Background:

Tuberculosis remains a global health threat, responsible for over a million deaths annually, despite extensive efforts for treatment. It is a highly infectious disease caused by *Mycobacterium tuberculosis*. It is characterized by necrotizing granulomas and inflammation primarily in the lung, but can manifest in other organs too [1]. Transmission takes place primarily through respiratory droplet infections. Following inhalation, the bacteria proliferates mainly in the well-ventilated regions of the upper lung, predominantly in the alveolar macrophages. The regimen for pulmonary tuberculosis includes two months of quadruple therapy. It consists of isoniazid, rifampicin, Ethambutol and pyrazinamide. This is immediately followed by 4 months of combined administration of isoniazid and rifampicin [2]. A major challenge associated with tuberculosis is antimicrobial drug resistance. According to the WHO, *Mycobacterium tuberculosis* is a major contributor of drug resistance, as it contributes to 29% of deaths caused by resistant infections [3]. Tuberculosis is a major health concern in India, making up for approximately 28% of the global tuberculosis incidence. It has a high burden, with ~31-41% prevalence in the country. In the current scenario, approximately two deaths occur every three minutes from Tuberculosis. The major problems in treating TB in India are the substandard health care infrastructure in rural districts. Unrestricted private health care leads to extensive inappropriate use of first- and second-line TB drugs [4]. Due to the urgent need for new therapeutic agents, targeting essential proteins involved in *mycobacterial* cell wall biosynthesis has become a promising approach. DprE1 is a validated target involved in the biosynthesis of arabinogalactan and lipoarabinomannan. They are key components of the *Mycobacterium tuberculosis* cell wall. D-arabinofuranose in these polymers is derived from decaprenylphosphoryl- D-arabinose (DPA). DPA is formed by epimerization of decaprenylphosphoryl-D-ribose (DPR). DprE1 catalyzes the oxidation of DPR to decaprenylphosphoryl-2'-keto-ribose (DPX) with simultaneous FAD reduction. DprE2 then reduces DPX to DPA and re-oxidizes FADH_2_. The periplasmic localization of DprE1 makes it an accessible and attractive drug target [5]. This study involves three Indian phytochemicals with traditional medicinal use and potential anti-tuberculosis activity. These include Withanolide D and Withaferin A from *Withania somnifera* and Nimbolide from *Azadirachta indica*. Withanolide D has been shown to bind effectively to *M. tuberculosis* PknG, a key bacterial kinase, indicating potential antimicrobial activity. Withaferin A has shown inhibition of drug resistant strains of *M. tuberculosis* by immunomodulation. Nimbolide exhibits antimicrobial properties [6]. Molecular docking of these compounds with DprE1 was performed using PyRx [7]. This was followed by 100 nanosecond molecular dynamics simulations using Schrödinger Desmond [8]. Finally, toxicity studies were conducted using Protox-3.0 to assess the toxicity profiles of each compound [9]. Alongside these phytochemicals, two standard first-line antibiotics have been included as reference molecules. Ethambutol targets *mycobacterial* cell wall synthesis, while Isoniazid inhibits mycolic acid production. This allows analysis profiles between DprE1 against phytocompounds and standard antibiotics. Therefore, it is of interest to evaluate the docking of Withanolide D, Withaferin A and Nimbolide with DprE1. Their binding affinities are compared with Ethambutol and Isoniazid to assess their therapeutic potential.

## Materials and Methods:

The crystal structure of DprE1 (PDB ID: 4FDN) was obtained from Protein Data Bank [10]. The structure was determined by X-ray diffraction at a resolution of 2.40 Å with an R-free value of 0.226. It is expressed in *Escherichia coli*. The protein was cleaned using UCSF Chimera [11] and water molecules and nonstandard residues were removed to simplify the structure. The active sites of DprE1 were identified from PDBsum [12] to define the docking region of the protein. The 3D structures of the phytochemicals and antibiotics were obtained from the PubChem database [13] in the SDF format. Ligand preparation, including conversion of files to PDBQT format and energy minimization was done using PyRx. The molecular docking was performed using the AutoDock Vina engine within PyRx. Active sites selected in Vina Wizard to define the docking region. The resulting protein-ligand complexes were visualized using UCSF Chimera to generate the combined structures. Discovery Studio Visualizer [14] was used to analyze hydrophobic interactions and hydrogen bonding.

## Molecular dynamics simulations:

Molecular dynamics simulations were performed for the DprE1 complexes with the selected phytochemicals and antibiotics using Desmond. It employed the OPLS_2005 force field for 100 ns (nanosecond) simulation time. The complexes were solvated in an orthorhombic box of 100 x 100 x 100 Å using the TIP3P water model and periodic boundary conditions were applied. To neutralize the system, counter ions (Na^+^ and Cl^-^) were added. System equilibration was performed following Desmond's standard protocol, involving restrained minimizations and short MD simulations. This allowed gradual relaxation of the protein backbone to prevent any structural distortions. The equilibrated systems were subjected to production runs with NPT at 300 K and 1.01325 bar for 100 ns. Long-range electrostatic interactions were computed using the particle-mesh Ewald method and a 9 Å cutoffs was used for van der Waals interactions. Trajectory analyses were conducted to assess the structural stability and dynamic behaviour of the DprE1-phytochemical and DprE1-antibiotic complexes. The root mean square deviation (RMSD) was calculated to evaluate the stability of the system. Root mean square fluctuation (RMSF) values were found to analyze the residue flexibility. The hydrogen bond occupancy and persistence were closely monitored throughout the simulation to gain insights into the stability of the complexes [15].

## Toxicity prediction:

To evaluate the safety profiles of the selected phytochemicals and antibiotics, toxicity prediction was performed using Protox-3.0. The SMILES structures of all compounds were submitted to the server to predict toxicity levels and all available endpoints were chosen. A range of toxicity endpoints were assessed, including hepatotoxicity, neurotoxicity, immunotoxicity and carcinogenicity. The toxicity profile for three phytochemicals Nimbolide, Withaferin A and Withanolide D, along with two antibiotics, Ethambutol and Isoniazid were predicted and analyzed.

## Results and Discussion:

Molecular docking was performed using PyRx software. The target protein, DprE1 (PDB ID: 4FDN) was chosen for docking. The phytochemicals, Nimbolide, Withaferin A and Withanolide D along with the antibiotics Ethambutol and Isoniazid were docked against the target protein. Among the tested compounds, Withanolide D had the strongest binding affinity of -10.6 kcal/mol. This was followed by Nimbolide and Withaferin A which showed binding affinities of -10.3 kcal/mol and -10.2 kcal/mol respectively. The binding of the antibiotics, Ethambutol and Isoniazid were comparatively weaker, with binding affinities of -5.3 kcal/mol and -5.7 kcal/mol respectively. As shown in Table 1 (see PDF), the lower binding energy values of the phytochemicals suggest stronger binding interactions and potential inhibitory effects against DprE1 compared to the antibiotics. The binding interactions of DprE1 with the selected phytochemicals and antibiotics were visualized using BIOVIA Discovery Studio Visualizer. This revealed hydrogen bonds, hydrophobic contacts and van der Waals interactions in Figure 1 (see PDF). Molecular dynamics simulation was done to analyze the internal motions of the receptor-ligand complex over time under flexible solvent conditions. To validate the binding of the complexes, simulations were performed using Desmond software. RMSD analysis over 100 ns indicated that all receptor-ligand complexes were generally stable, with minor fluctuations. Among the phytochemicals, the DprE1-Nimbolide complex showed the greatest protein stability around 0.8 Å until 90 ns before rising to 3.2 Å, while the ligand increased from 1.6 to 5.6 Å. In the DprE1-Withaferin A complex, protein and ligand RMSD rose gradually from 0.8 to 2.8 Å and 0.4 to 2.8 Å respectively. The DprE1-Withanolide D complex remained stable with protein RMSD from 0.75 to 1.75 Å and ligand fluctuating between 1.0 and 4.5 Å. For the antibiotics, the DprE1-Ethambutol complex exhibited an increase in protein RMSD from 1.6 to 2.8 Å and the ligand RMSD from 1.8 to 3.6 Å. In the DprE1-Isoniazid complex, the protein RMSD rose from 1,2 to 2,4 Å and the ligand RMSD decreased from 2.4 to 1.5 Å. The RMSD profile for the DprE1-Nimbolide complex is shown in Figure 2 (see PDF).

The RMSF analysis revealed that all DprE1-ligand complexes exhibited minimal fluctuations, indicating overall structural stability during simulation. Among the phytochemicals, the DprE1-complex showed the lowest average RMSF values. They were around 1.0 Å with peaks around residues 250-270 and 290-310, up to 4.5 Å. The DprE1-Withaferin A complex displayed moderate fluctuations with RMSF around 1.2 Å and peaks at residues 230, 260 and 270 reaching 4.8 Å. The DprE1-Withanolide D complex had an average of 1.0 Å with a peak at residue 270, reaching 4.0 Å. Among the antibiotics, the DprE1-Ethambutol complex showed higher flexibility, with peaks at residues 0-20 (4.1 Å) and 250-300 (5.4 Å). The DprE1-Isoniazid complex was relatively stable, showing an average of 1.0 Å with peaks at residues 260-300 and at 350 (4.5 Å). Overall, the lower RMSF values for the DprE1-Nimbolide complex indicates stronger stability and binding interactions compared to the other phytochemicals. Protein-ligand contact analysis was performed to assess the stability of interactions between the receptor and ligand during the molecular dynamics simulation. The interaction fractions indicate the proportion of simulation time each residue participates in hydrogen bonds, hydrophobic contacts, ionic interactions, and water bridges. The results showed that Lys418 had the highest interaction fraction (~0.6), mainly through water bridges, indicating a key role in ligand stabilization. Tyr60 also showed strong interactions (~0.4), primarily hydrophobic, suggesting its contribution to a stable binding environment. Gln334 exhibited notable hydrogen bonding and water-mediated interactions, highlighting its importance in binding stability. Other residues such as Asn369, Val365, and Trp230/Trp323 contributed moderate interactions, while ionic interactions were minimal. Overall, the complex is stabilized by a combination of hydrophobic contacts, hydrogen bonds, and water bridges, with Lys418, Tyr60, and Gln334 playing major roles Figure 3 (see PDF) & Figure 4 (see PDF).

The toxicity profiles of the selected phytochemicals (Nimbolide, Withaferin A, Withanolide D) and antibiotics (Ethambutol and Isoniazid) were predicted using Protox-3.0. The toxicity levels were divided into 6 classes based on their median lethal dose LD50: class 1 (≤5 mg/kg): fatal if swallowed, class 2 (5-50 mg/kg): fatal if swallowed, class 3 (50-300 mg/kg): toxic if swallowed, class 4 (300-2000 mg/kg): harmful if swallowed, class 5 (2000-5000 mg/kg) may be harmful if swallowed and class 6 (>l5000): non-toxic. Nimbolide exhibited toxicity class 5 with predicted LD50 of 1000 mg/kg. Withaferin A and Withanolide D were both found to fall under toxicity class 3 with an LD50 value of 300 mg/kg. All three phytochemicals were predicted to be immunotoxic. Ethambutol was classified as toxicity class 4 with an LD50 of 998 mg/kg and predicted to cause respiratory toxicity. Isoniazid was found to be under toxicity class 3 with an LD50 of 133 mg/kg and predicted to cause carcinogenicity and hepatotoxicity. The summarized LD50 values with predicted toxic effects are presented in Table 2 (see PDF). Overall, the phytochemicals demonstrated a comparatively lower toxicity level, suggesting their potential safety for further studies. The present study evaluated the binding potential of three phytochemicals- Nimbolide, Withaferin A and Withanolide D against the essential *mycobacterial* enzyme DprE1. It also compared their interactions with standard antibiotics - Ethambutol and Isoniazid. Overall, Nimbolide demonstrated the strongest inhibitory potential with a binding affinity of -10.3 kcal/mol. Molecular simulation studies further validated the stability of the DprE1-Nimbolide complex. It was shown to have low RMSD and RMSF values indicating minimal fluctuations in the protein backbone and ligand position over 100 ns. Compared to standard antibiotics, Nimbolide exhibited a stronger binding and more stable interactions, suggesting a superior inhibitory potential against DprE1. Toxicity predictions indicated that Nimbolide has a favourable safety profile (LD50=1000 mg/kg, Class 4), supporting its potential as a safe therapeutic. Previous studies have also supported the antimicrobial and anti-tubercular properties of Nimbolide. It demonstrated the anti-tubercular activity in an animal model of tuberculous arthritis [16]. It was also found to have strong molecular docking interactions with microbial protein targets, reinforcing its broad antimicrobial potential [17]. Recent studies have further highlighted the effectiveness of phytochemicals as potential DprE1 inhibitors through stable binding interactions and favorable pharmacokinetic properties, supporting the role of natural compounds as promising alternatives in anti-tuberculosis drug development [18]. Thus, Nimbolide is a promising phytochemical for anti-tuberculosis therapy, combining structural stability, binding affinity and a relatively safe toxicity profile.

## Conclusion:

Nimbolide is a promising anti-tuberculosis phytochemical demonstrating strong binding affinity and stable interactions with DprE1. Compared to standard antibiotics, its favorable toxicity profile suggests potential safety for therapeutic use. These results position Nimbolide as a leading candidate for further experimental validation and development as a novel anti-tuberculosis agent.

## Disclosure statement:

No potential conflict of interest was reported by the author(s).

## Funding:

No funding was received.

## Ethical approval:

Not applicable.

## Informed consent:

Not applicable.
